# Supplementary material for: DPA4 Suppresses Adventitious Root Formation via Transcriptional Regulation of CUC2 and ULT1, Decreasing Auxin Biosynthesis in Arabidopsis Leaf Explants
Source: Int J Mol Sci. 2025 Nov 24;26(23):11336. doi: 10.3390/ijms262311336 (PMC12692053; doi:10.3390/ijms262311336)
Supplement: Supplementary file 1 [file ijms-26-11336-s001.zip › ijms-3934550-supplementary.pdf]

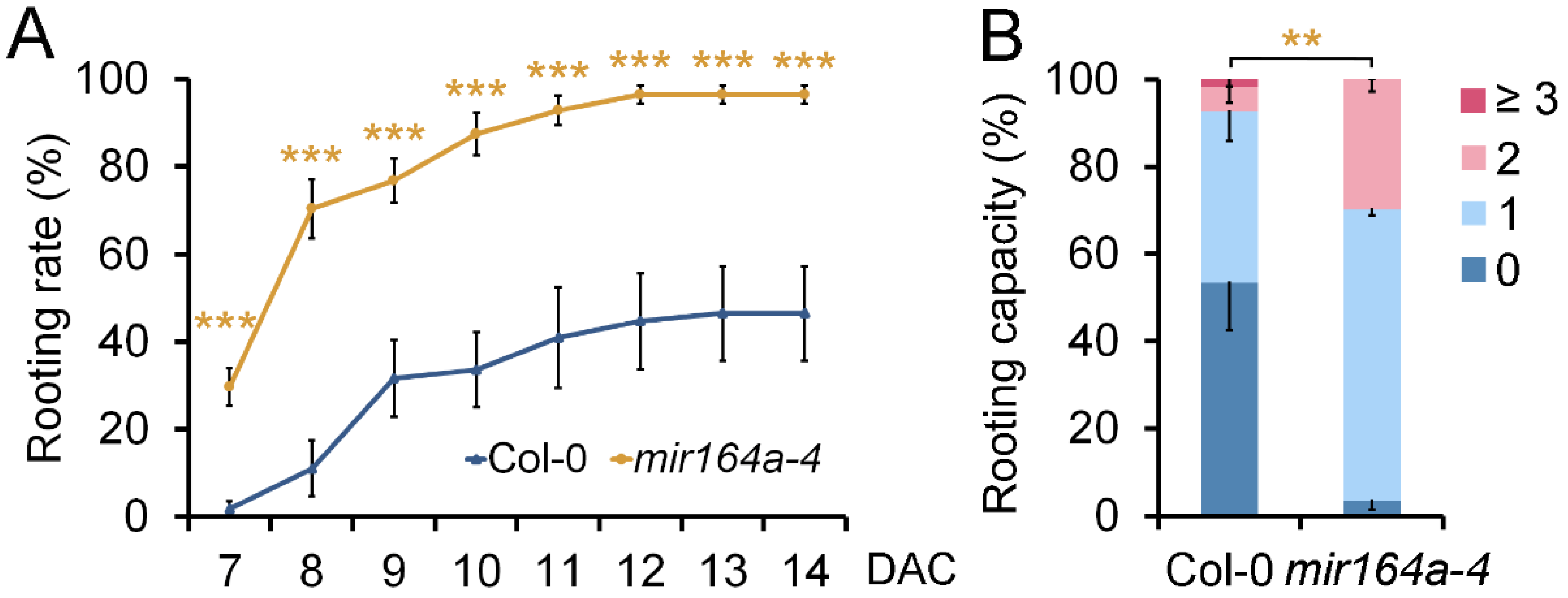

Supplementary Figure S1. DNRR is increased in *mir164a-4* compared to wild-type. (A) Rooting rate analyses of leaf explants from Col-0 and *mir164a-4* mutants in a time course. (B) Rooting capacity of leaf explants from Col-0 and *mir164a-4* cultured on B5 medium for 14 days (14 DAC). 0, 1, 2 and ≥3 represent AR numbers per leaf explant. Average values are shown (N ≥ 40 leaves from 5 individual plate for each single experiment), ± SEM. Statistical significance was determined by Student's *t*-test: ** $p < 0.01$ and *** $p < 0.001$.

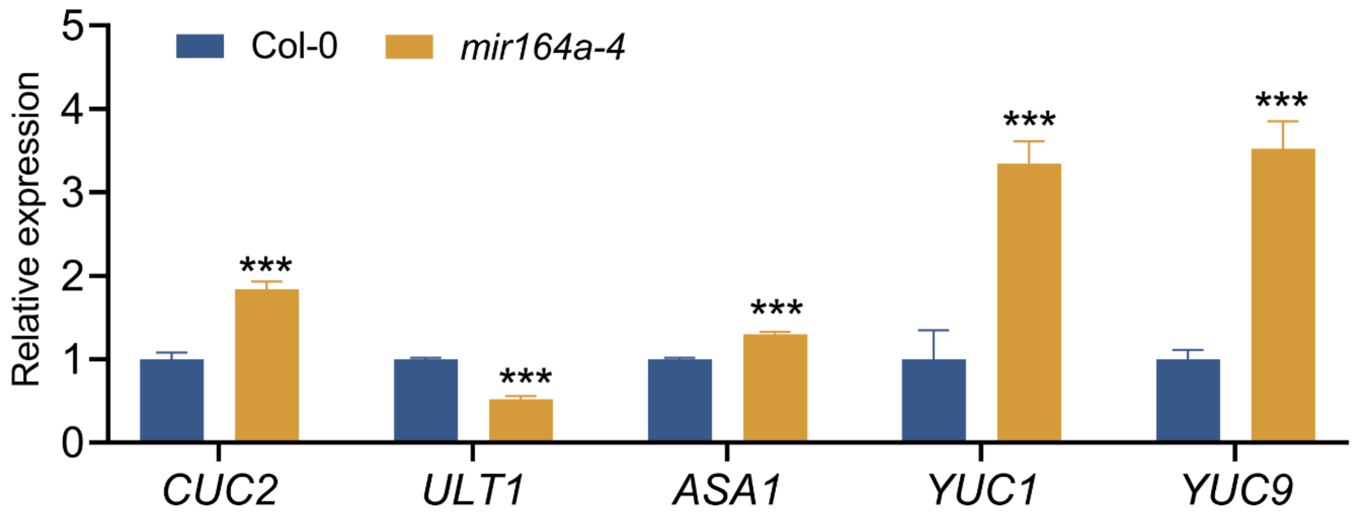

Supplementary Figure S2. Gene expression changes in *mir164a-4* leaf explants. RT-qPCR analysis of gene expression levels in Col-0 and *mir164a-4* leaf explants, 4 hours after detachment on B5 medium (4 HAC). Average values are shown (N = 4), ± SEM. Statistical significance was determined by Student's *t*-test: *** $p < 0.001$.

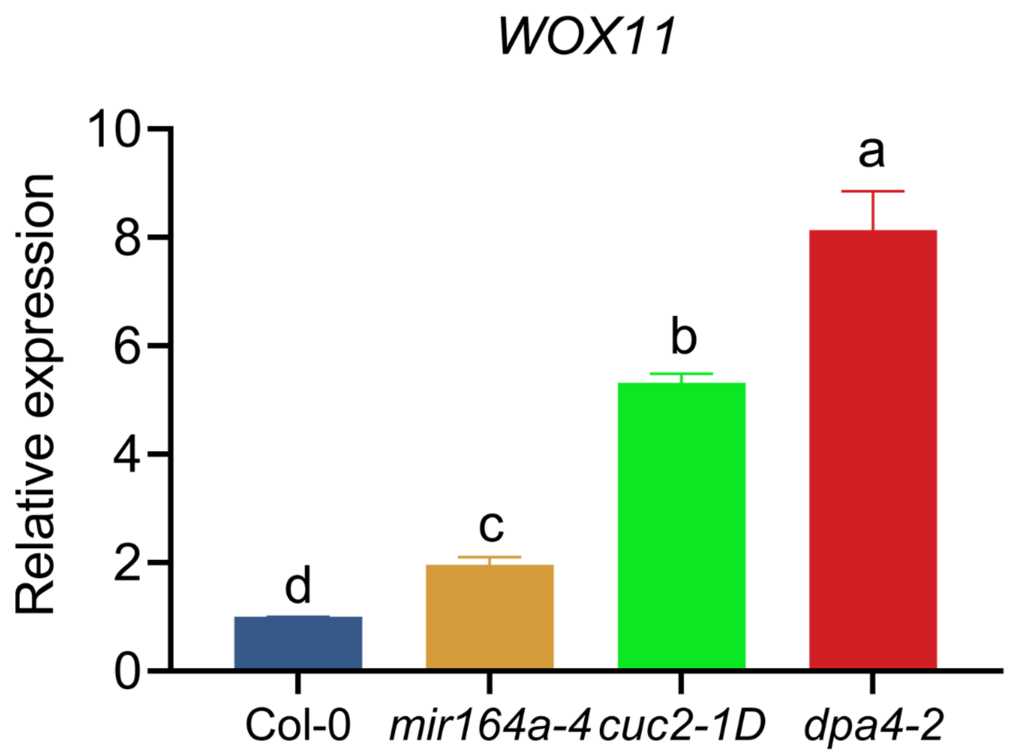

Supplementary Figure S3. *WOX11* expression levels in *mir164a-4, cuc2-1D and dpa4-2* leaf explants. RT-qPCR analysis of *WOX11* expression levels in Col-0, *mir164a-4, cuc2-1D and dpa4-2* leaf explants, 2 days after detachment on B5 medium (2 DAC). Average values are shown (N = 4), ± SEM. Statistical significance ($p \leq 0.05$) was determined by one way ANOVA and Duncan's LSD and a-d mark groups of significant differences.

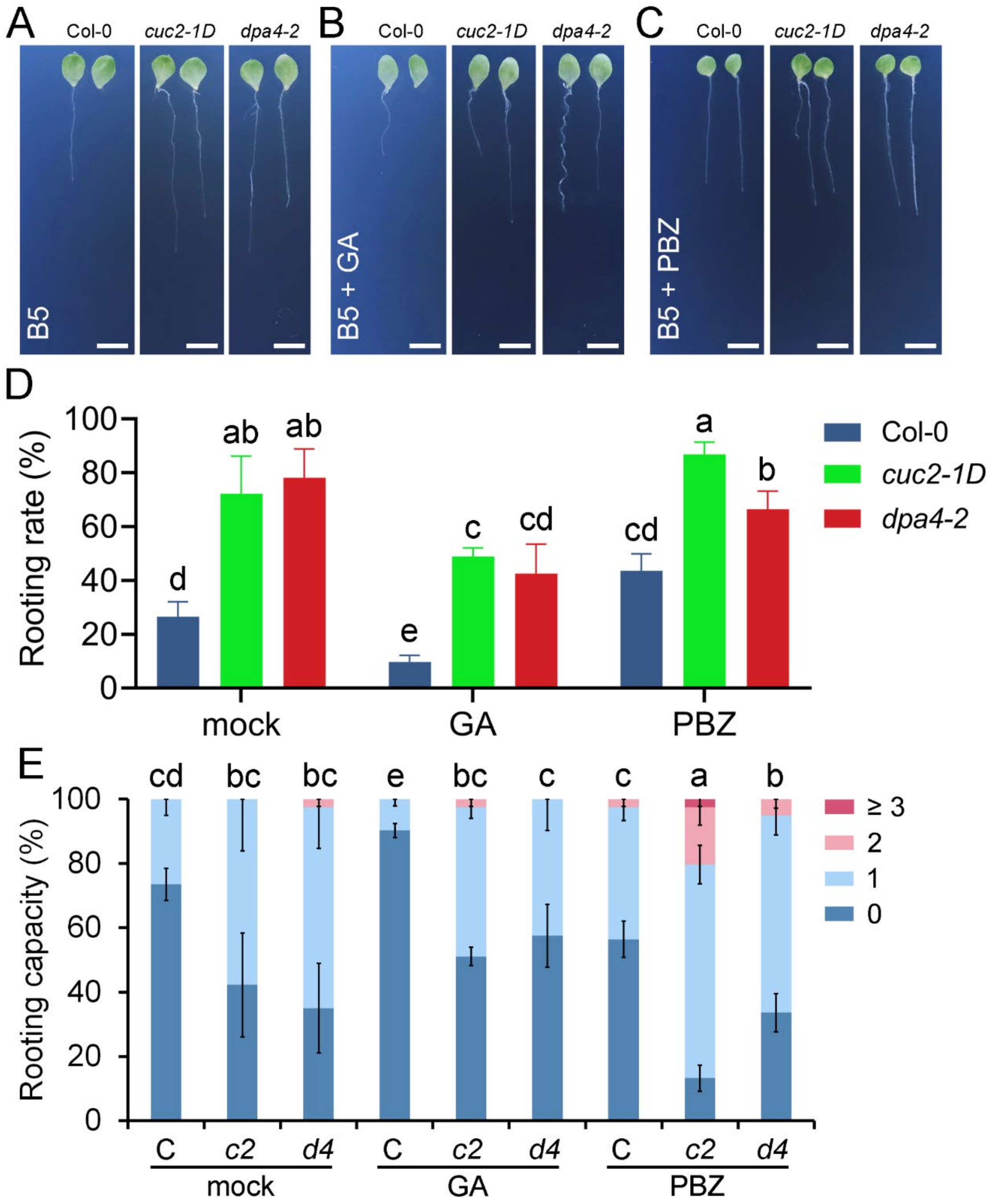

Supplementary Figure S4. Effects of GA and PBZ treatment on rooting rate and rooting capacity in leaf explants in darkness.

(**A-C**) Leaf explants from Col-0, *cuc2-1D*, and *dpa4-2* cultured on B5 medium without (**A**, mock) or with GA (**B**) or PBZ (**C**), treatment, scale bars indicate 500 μm.

(**D**) Rooting rate of leaf explants from Col-0, *cuc2-1D*, and *dpa4-2* with and without GA and PBZ treatment.

(**E**) Rooting capacity of leaf explants from Col-0 (C), *cuc2-1D* (*2c*), and *dpa4-2* (*d4*) with and without GA and PBZ treatment. 0, 1, 2 and ≥3 represent AR numbers per leaf explant. Average values are shown (N ≥ 40 leaves from 5 individual plate for each single experiment), ± SEM. Statistical significance was determined by Student's t-testa-d mark groups of significant differences ($p \leq 0.05$).

Supplementary Table S1: Primer pairs for RT-qPCRs

Primer name	Primer sequence (5'→3')
qRT-eIF4A Fw	CGTGGTTTCAAGGACCAGA
qRT-eIF4A Rv	TGCGGAGAACACACCAACT
qRT-CUC2 Fw	GCTCCAAGGATGAATGGGTG
qRT-CUC2 Rv	ATGAGTTAACGTCTAAGCCCA
qRT-ULT1 Fw	CGATGAAGACAAGTTGACACC
qRT-ULT1 Rv	TACCACAATCGTTACACCCA
qRT-ERF109 Fw	CTTATGATCGAGCCGCGATT
qRT-ERF109 Rv	TCCTCCGTTCCATTGCTCTG
qRT-ABR1 Fw	CACAACCTGTGCACCAAACC
qRT-ABR1 Rv	GGGCAAAAGGGTAGTCGTTGA
qRT-ASA1 Fw	ATGTCTTCCTCTATGAACGTAGC
qRT-ASA1 Rv	ACAGCGGTAAATTGGTATAAGG
qRT-YUC1 Fw	CGATGTCGGAGCTATGTCTC
qRT-YUC1 Rv	CTGTACAAGTTTATTACTTCG
qRT-YUC4 Fw	GACAAATTAAAGTGACGCAAGCC
qRT-YUC4 Rv	GTTCCCGAAAGTCCTCTTCTC
qRT-YUC6 Fw	GGTAGTTAAGCACACGTGTC
qRT-YUC6 Rv	GGCTAGCGTGCCAACGTCGAG
qRT-YUC9 Fw	TAGAACAACCTCAGACGGAGAG
qRT-YUC9 Rv	AAACATGAACCGAGCTTCTAACG
qRT-WOX11 Fw	CGCAACCACCAACACTTGTGACC
qRT-WOX11 Rv	CCTGAGGAATGCACCAAAACC
